# The Portuguese short version of the Eating Disorder Examination Questionnaire: Validity and Reliability in men across multiple ages

**DOI:** 10.1192/j.eurpsy.2024.1171

**Published:** 2024-08-27

**Authors:** A. Silva, A. Macedo, C. Marques, M. J. Brito, A. T. Pereira, C. Coelho

**Affiliations:** ^1^Faculty of Medicine, University of Coimbra, Coimbra, Portugal; ^2^Institute of Psychological Medicine, Faculty of Medicine, University of Coimbra, Coimbra, Portugal

## Abstract

**Introduction:**

The Eating Disorder Examination Questionnaire short version (EDE-Q7) presented better psychometric properties than the Fairburn’s 28-items original version, not only in girls (Machado et al. 2018), but also in older women (Pereira et al. 2021; Pereira et al. 2022). It comprises 7 items in three subscales: Dietary Restraint/DR; Shape and Weight Overvaluation/SWO and Body Dissatisfaction/BD. In a more recent clinical study in men (Laskowski et al. 2023) the factors associated with body concerns and dissatisfaction weren’t fully represented in the questionnaire, possibly indicating differences in body ideals, specially relating to musculature.

**Objectives:**

We aimed to analyze the psychometric properties of the Portuguese version of EDE-Q7 in males.

**Methods:**

Participants were 227 male individuals with a mean age of 30.41 years (±13.96; range: 14-73 years). They answered an online survey including the Portuguese versions of the Screen for Disordered Eating/SDE; the Body Image Concern Inventory/BICI and the Muscle Dysmorphia subscale of the Eating Disorder Assessment for Men/DM-EDAM.

**Results:**

Confirmatory Factor Analysis showed that the second order model presented good fit (χ^2^/df=2.437; RMSEA=.0794; CFI=.986 TLI=.974, GFI=.967). Cronbach’s alpha was .856 for the total, .876 for DR and .917 for SWO and .900 for BD. All items contributed to internal consistency and presented high internal validity. Pearson’s correlations of EDE-Q7 with BICI (.465), DM-EDAM (.384) and SDE (.361) were significant (p<.001) and moderate.

**Conclusions:**

Also in men, the Portuguese version of EDE-Q7 demonstrates good validity (construct and convergent) and reliability.

**Disclosure of Interest:**

None Declared

